# A Comparative Analysis of the SARC-F Questionnaire and the Malnutrition–Inflammation Score for Sarcopenia Risk Assessment and Negative Outcome Probability in Chronic Hemodialysis Patients

**DOI:** 10.3390/jcm13185554

**Published:** 2024-09-19

**Authors:** Lea Katalinic, Ivana Juric, Vesna Furic Cunko, Vedran Premuzic, Bojan Jelakovic, Nikolina Basic-Jukic

**Affiliations:** Department of Nephrology, Arterial Hypertension, Dialysis and Transplantation, University Hospital Centre Zagreb, 10000 Zagreb, Croatia

**Keywords:** protein–energy wasting, sarcopenia, hemodialysis, SARC-F, MIS, outcome

## Abstract

**Background/Objectives**: Protein–energy wasting (PEW) and sarcopenia are common in chronic hemodialysis (HD) patients, leading to numerous complications and increased mortality. This study aimed to compare the reliability of the SARC-F (Strength, Assistance in walking, Rise from a chair, Climb stairs, and Falls) and the Malnutrition–Inflammation Score (MIS) in assessing sarcopenia and predicting negative outcomes in HD patients. **Methods**: This cross-sectional study enrolled 109 HD patients. Nutritional assessments were performed, and blood samples were taken for routine blood laboratory investigations. The MIS was used as a scoring system to represent the severity of PEW, while the SARC-F was applied as an indicator of sarcopenia risk and general functional capacity. A multivariable logistic regression was conducted to analyze the association of several predictors with a negative cross-sectional outcome (death). **Results**: Patients with SARC-F scores ≥ 4 and MISs ≥ 6 were older, had significantly lower albumin and prealbumin levels, and more severe anemia. They were also more likely to report weight loss and poor appetite. A higher MIS was closely associated with unfavourable nutritional status according to the International Society of Renal Nutrition and Metabolism (ISRNM) criteria for PEW. However, in 71.25% of patients with satisfactory functional capacity (SARC-F scores 0–3), some form of PEW was still observed. After performing logistic regression modelling, only the MIS remained strongly associated with the probability of a negative outcome. **Conclusions**: The SARC-F alone often did not correspond to an increased sarcopenia risk or clear clinical and biochemical indicators of PEW in HD patients. When assessing nutritional risk in this group, it is recommended to use more detailed tools, such as the MIS, to ensure the accurate identification of those at the highest risk for negative outcomes.

## 1. Introduction

Protein–energy wasting (PEW) is a process characterized by the continuous loss of muscle mass and visceral adipose tissue, leading to a significant reduction in overall energy reserves. It is considered one of the late complications of chronic kidney disease (CKD) [1]. PEW is a dynamic process that eventually leads to sarcopenia, which is a decrease in skeletal muscle mass and strength, resulting in poor physical performance [2]. Although sarcopenia is traditionally associated with aging (primary sarcopenia), it can also be disease-related, regardless of age (secondary sarcopenia). In fact, chronic disease-related muscle loss tends to be more progressive, non-linear, and of a considerably greater degree [3,4].

PEW and concomitant sarcopenia result in cachexia, muscle weakness (dynapenia), and frailty, leading to infectious and cardiovascular (CV) complications, functional dependence, and a low quality of life (QoL) [5,6,7]. Because of its importance in maintaining metabolic and functional stability, the preservation of muscle mass is crucial in the management of PEW in CKD patients [8].

There is no perfect tool to easily identify dialysis patients at risk for nutritional derangements and muscle wasting. Therefore, a combination of different methods is used when performing nutritional screening, including clinical, biochemical, and nutritional parameters [9]. Several tools have been developed to quickly and easily assess nutritional risk, predominantly prediction scores validated specifically for hemodialysis (HD) patients, such as the Malnutrition–Inflammation Score (MIS) and the Subjective Global Assessment (SGA) which combine all these criteria [10,11]. The SARC-F (Strength, Assistance in walking, Rise from a chair, Climb stairs, and Falls) is a questionnaire that estimates the presence of sarcopenia risk and sarcopenia-related functional impairments in a self-reported manner. A SARC-F score ≥ 4 is considered highly predictive of the development of sarcopenia and poor treatment outcomes [12].

The aim of our study was to compare the reliability of the SARC-F and MIS in assessing sarcopenia and predicting negative outcomes in patients on chronic hemodialysis (HD).

## 2. Materials and Methods

This descriptive cross-sectional study was conducted at the dialysis unit of the University Hospital Centre (UHC) Zagreb. This study was approved by the Medical Ethics Committee (protocol code 8.1.-14/121-2, number: 02/21/JG, approval date: 25 February 2015). Patients were eligible if they had been on HD for at least 6 months and were aged ≥18 years.

Demographic data were recorded from patients’ medical files, including age, gender, comorbidities, primary kidney disease, and HD vintage. Blood samples were collected before a midweek HD session for routine laboratory investigations, which included complete blood count values, creatinine, urea, electrolytes, total protein levels, albumin, prealbumin, ferritin, lipid profile, and C-reactive protein (CRP) levels.

Nutritional assessment involved a clinical examination using the MIS, which served as a scoring system for the severity of PEW. Patients with an MIS ≥ 6 were classified as malnourished. The SARC-F was used to assess sarcopenia risk and general functional capacity, with a cutoff point of ≥4 indicating sarcopenia. Dry weight and height were recorded to calculate body mass index (BMI). According to the International Society of Renal Nutrition and Metabolism (ISRNM) criteria, patients were categorized based on BMI as follows: <19 kg/m^2^—severe malnutrition; 19–21.9 kg/m^2^—moderate malnutrition; 22–24 kg/m^2^—mild malnutrition; 24.1–30 kg/m^2^—normal nutritional status; and >30 kg/m^2^—obesity. Among the obese patients, those with signs of muscle loss were identified as having sarcopenic obesity. The ISRNM criteria for diagnosing PEW were applied, which include four main categories: biochemical criteria, low BMI, reduced total body fat or weight loss, decreased muscle mass, and low protein or energy intake. Patients meeting 3 out of these 4 criteria were classified as malnourished [1].

### Statistical Analysis

Statistical analysis was performed using Stata/SE 11.2 for Windows (StataCorp LLC., College Station, TX, USA). Categorical variables were expressed as frequencies and percentages. The normality of continuous variables was tested using the Shapiro–Wilk test. Continuous data were described by the median and interquartile range (IQR). Pearson’s χ^2^ test (or Fisher’s exact test if any expected cell frequency in a contingency table was ≤5) was used to analyze differences between proportions. The Mann–Whitney U test was employed to compare medians between two groups.

To identify significant predictors of the negative cross-sectional outcome (death), we initially performed univariate analysis. The variables assessed included demographic characteristics (i.e., age, gender, primary kidney disease, HD vintage), clinical characteristics (i.e., presence of diabetes, arterial hypertension (AH), cardiovascular disease (CVD), BMI, MIS, SARC-F, weight loss, reduced appetite), and biochemical parameters (i.e., creatinine, CRP, hemoglobin, electrolytes, albumin, prealbumin, and lipid panel levels). Variables with *p*-values < 0.05 (MIS, CRP levels) were considered significant and were included in the subsequent multivariate logistic regression model for in-depth analysis. A multivariable logistic regression was conducted using the Stepwise method. This model was adjusted for age, comorbidities (diabetes, CVD, AH), HD vintage, albumin, and CRP levels to assess the independent contributions of these predictors to mortality.

## 3. Results

There were 67 male (61.5%) and 42 female (38.5%) patients, with a median age of 61 years (IQR: 27–85). Primary kidney disease was diabetic nephropathy in 28.4% of patients, followed by chronic glomerulonephritis (27.5%) and hypertensive kidney disease (16.5%). The median time spent on HD was 50 months (IQR: 6–168), with a minimum treatment time of 3 h, three times a week. All patients were treated with bicarbonate HD, high-flux polysulfone dialyzers, and ultrapure dialysate with a flow rate of 500 mL/min. Among the observed comorbidities, 29 patients (26.9%) had diabetes, 42 (39%) had CVD, and 83 (77%) had AH.

The median MIS was 7 (IQR: 5–21), with a score ≥ 6 in 72.5% of patients. The median SARC-F score was 1 (IQR: 0–10), with a score ≥ 4 in 26.6% of patients. According to the ISRNM criteria, 21.1% of patients had normal nutritional status, 29.4% were mildly malnourished, 23.9% were moderately malnourished, and 5.5% were severely malnourished. Among the obese patients, 20.2% had signs of muscle wasting and were classified as having sarcopenic obesity.

Patients in the lowest BMI group were all classified as moderately or severely malnourished (*p* < 0.001) with a median MIS of 12 (IQR: 9–14; *p* = 0.04). In contrast, patients in the other BMI groups were more likely to have either normal nutritional status or to be mildly to moderately malnourished. Notably, overweight or obese patients demonstrated significantly higher SARC-F scores (*p* = 0.01) and were more likely to have comorbid diabetes (*p* = 0.02) and AH (*p* = 0.02). The baseline characteristics of the cohort are shown in Table 1.

Patients with diabetes were older (*p* = 0.001), had a shorter dialysis vintage (*p* = 0.004), and were more likely to have CVD (*p* < 0.001). Despite having a higher BMI (*p* = 0.006), they exhibited lower albumin and prealbumin levels (*p* = 0.001; *p* = 0.01) and had worse scores for predicting PEW and sarcopenia (SARC-F: *p* = 0.008; MIS: *p* = 0.01). Consequently, the incidence of sarcopenic obesity was significantly higher in diabetic patients. In contrast, the non-diabetic group more frequently maintained a normal nutritional status or showed only a mild form of PEW (*p* = 0.002; see Table 2).

When divided into two groups based on the SARC-F score (0–3 or ≥4), a SARC-F score of ≥4 was associated with older age (<0.001), decreased hemoglobin and calcium levels (*p* = 0.02; *p* = 0.03), lower albumin and prealbumin levels (*p* = 0.002; *p* < 0.001), and a higher MIS (*p* = 0.001). Despite having a higher BMI, this subgroup more frequently exhibited signs of moderate-to-severe PEW and sarcopenic obesity (*p* < 0.001). They were also more likely to report weight loss (*p* = 0.003) and poor appetite (*p* = 0.01; see Table 3 and Table 4). Notably, 71.25% (*n* = 57) of patients with satisfactory functional capacity (SARC-F 0–3) showed some form of PEW.

Patients with an MIS ≥6 were older (*p* = 0.001) and had lower levels of albumin, prealbumin, and total serum protein (*p* < 0.001; *p* < 0.001; *p* = 0.03), as well as more pronounced chronic anemia parameters, including lower hemoglobin and total iron-binding capacity (TIBC) levels (*p* < 0.001; *p* = 0.02). They were also more likely to have diabetes (*p* = 0.03), CVD (*p* = 0.02), and exhibited poorer functional capacity, with a median SARC-F score of 1 (IQR: 0–6) (*p* < 0.001). A higher MIS was strongly associated with an unfavourable nutritional status according to the ISRNM criteria for PEW (*p* < 0.001), with marked weight loss and appetite issues (*p* = 0.003; *p* < 0.001; see Table 5 and Table 6).

Finally, we examined whether either of the evaluated scores could serve as an independent predictor of poor treatment outcomes and death. Within four months following the initial evaluation, seven patients (6.42%) died, with deaths primarily due to CVD (*n* = 5) and infectious causes (*n* = 2). Deceased patients had a significantly higher MIS (median MIS 12 (IQR: 9–13); *p* = 0.001) and more severe signs of PEW according to the ISRNM criteria (*p* = 0.001).

In univariate logistic regression analysis, MIS and CRP levels were identified as significant predictors of a negative outcome (see Table 7).

Logistic regression analysis, adjusted for age, comorbidities, HD vintage, albumin and CRP levels, revealed that the MIS was the only score independently associated with mortality risk (odds ratio [OR] = 1.59; χ^2^ = 16.2; *p* = 0.04). This finding indicates that the MIS is a robust independent predictor of adverse outcomes in HD patients, explaining 37% of the variance in negative outcomes (as per Negelkerke R^2^) and accurately classifying 93% of cases (see Table 8).

## 4. Discussion

In our cohort of 109 HD patients with a median age of 61 years, malnutrition and inflammation, defined by an MIS cutoff of ≥6, were present in 72.5% of patients—a prevalence considerably higher than previously reported rates of 28–54% [13,14]. A SARC-F score of ≥4, indicating significant sarcopenia risk, was observed in 26.6% of patients, aligning with Yamamoto’s finding of 26.7% in HD patients [15] and similar to distributions reported by Lin et al. and Imamura et al. [16,17].

Patients with SARC-F scores ≥ 4 and MISs ≥ 6 were older and had significantly lower albumin and prealbumin levels, as well as more severe anemia. Patients with diabetes exhibited worse nutritional outcomes, with lower levels of albumin and prealbumin, higher MIS and SARC-F scores, and a higher prevalence of sarcopenic obesity compared to non-diabetic patients. A higher MIS was closely associated with unfavourable nutritional status. One notable finding is that even among patients with preserved functional capacity (SARC-F scores 0–3), 71.25% still exhibited some form of PEW, underscoring the need for more comprehensive nutritional assessment, particularly in dialysis patients with subtle functional declines.

Finally, after performing logistic regression modelling, only the MIS remained strongly associated with the probability of a negative outcome, highlighting its role as a key predictor in this population.

The high prevalence and progressive nature of muscle wasting in CKD are associated with significant clinical consequences including frailty, poor responsiveness to erythropoiesis-stimulating agents (ESAs), reduced QoL, and increased hospitalization and mortality risk. Therefore, routine nutritional assessment in CKD patients is strongly recommended [16,18,19,20]. The early detection of PEW allows for timely intervention, hence preventing further deterioration [21]. Although no consensus exists on the optimal screening tool, several validated questionnaires, such as the SGA, MIS, and ISRNM PEW criteria, are commonly used. However, these tools require trained personnel, making them less feasible and much more time-consuming compared to simpler tools like the Geriatric Nutritional Risk Index (GNRI) or the SARC-F, which are quicker and easier to administer [22].

The SARC-F, originally designed to assess sarcopenia risk in community-dwelling elderly populations, is increasingly being investigated in CKD patients [15,23,24,25,26,27,28,29]. Our study aimed to compare the reliability of the SARC-F and MIS in assessing sarcopenia and predicting adverse outcomes in HD patients. To our knowledge, this dual comparison has not been widely explored in previous studies, especially in the context of PEW in CKD, adding important insights to the growing body of the literature on effective nutritional screening tools in this population.

Yamamoto’s group was the first to highlight the SARC-F’s effectiveness in the dialysis population, associating it with impaired physical functioning and an increased risk of physical limitations. However, studies that followed have shown mixed results, suggesting that the SARC-F alone may be inadequate for screening sarcopenia in HD patients. Imamura et al. reported a sensitivity of 42.9% and specificity of 70.8% for the SARC-F in detecting sarcopenia, with 23.4% of patients with sarcopenia being missed when using the SARC-F alone [17]. This indicates that while the SARC-F can be useful for identifying muscle strength and functional impairments, it may miss cases of sarcopenia among HD patients.

The MIS emerged as a significant predictor of adverse outcomes, particularly mortality [11,22,30,31]. PEW is a complex syndrome characterized by inadequate dietary intake, increased nutritional losses during dialysis, and chronic inflammation, all of which contribute to muscle breakdown and further deterioration in nutritional status [1,32,33]. This vicious cycle of malnutrition, inflammation, and muscle wasting significantly impacts treatment outcomes, highlighting the need for effective screening tools [31,33,34].

Our survival analysis revealed that only the MIS was a significant independent predictor of mortality, consistent with previous studies linking malnutrition and inflammation to adverse outcomes in CKD patients [11,22,30,31]. The MIS is a comprehensive scoring system that integrates clinical, biochemical, and nutritional parameters, which is crucial given the multifaceted nature of PEW. It is tailored to CKD and incorporates all PEW criteria, making it a valuable tool for identifying patients at risk for poor treatment outcomes [11,31,35]. While the SARC-F helps assess sarcopenia risk and functional impairments, it may not fully capture the extent of PEW, particularly in patients with preserved functional capacity but significant nutritional deficits [17].

Several confounding factors known to influence outcomes in HD patients were accounted for in the analysis. Age is a critical factor, as older patients typically experience a higher prevalence of malnutrition, sarcopenia, and inflammation, which negatively impact survival rates [36,37,38]. Comorbidities such as diabetes and CVD are highly prevalent among HD patients and have been repeatedly associated with increased inflammation, worse nutritional status, and higher mortality [39,40,41,42,43,44]. Arterial hypertension further exacerbates cardiovascular risk, contributing to adverse outcomes [42,43,44]. Dialysis vintage also plays a significant role, with longer dialysis exposure linked to higher inflammation and deteriorating nutritional status [45]. Albumin levels, commonly used as a marker of nutritional status, are independently associated with mortality in HD patients, reflecting both malnutrition and inflammation [46,47,48]. Elevated CRP levels are a hallmark of systemic inflammation and have been shown to correlate with higher mortality, emphasizing the impact of inflammatory processes on patient outcomes [47,48]. Even after adjusting for these key confounders, the association between the MIS and mortality remained significant, suggesting that the MIS is a reliable independent predictor of adverse outcomes in HD patients. This finding is supported by Yamada et al., who demonstrated that the MIS is the gold standard for evaluating other simpler scoring systems [49].

Du et al. investigated the validity of the SARC-F questionnaire for screening sarcopenia among CKD patients. Among the 105 non-dialysis-dependent CKD patients and 125 HD patients, the prevalence of sarcopenia was 5.7 and 31.2%, while the sensitivity and specificity of the SARC-F were 16.7 and 98% for non-dialysis patients and 48.7 and 89.5% for dialysis patients, respectively [50]. In a meta-analysis of 29 studies which included 21,855 individuals (mean age of 63.3 ± 14.6 years, 38.7% males) among community-dwelling, geriatric inpatient, geriatric outpatient, nursing home, and long-term care populations, the SARC-F had low-to-moderate sensitivity (28.9–55.3%) and moderate-to-high specificity (68.9–88.9%) according to the European Working Group on Sarcopenia in Older People (EWGSOP; *n* = 13), revised EWGSOP 2019 definition (*n* = 6), Asian Working Group for Sarcopenia (AWGS; *n* = 13), Foundation for the National Institutes of Health (FNIH; *n* = 8), International Working Group on Sarcopenia (*n* = 9), and Society on Sarcopenia, Cachexia and Wasting Disorders (SCWD; *n* = 2) [51].

These findings suggest that while the SARC-F may serve as an initial screening tool, its effectiveness could be enhanced when used in combination with more objective assessment measures (i.e., physical performance tests such as gait speed, chair stand tests, and handgrip strength and body composition measures such as body composition monitoring). Given the findings from Yamamoto et al. in dialysis patients [17], but also the findings from other patients’ groups [50,51,52,53,54], all indicating a low-to-moderate sensitivity and specificity of the SARC-F, this approach may prevent the underestimation of sarcopenia in HD patients.

Our study has several limitations. First, the observational period for negative clinical outcomes was relatively short. The limited follow-up period may have influenced the results and reduced the ability to fully capture long-term outcomes related to malnutrition and sarcopenia. Consequently, the findings should be interpreted with caution, as a longer follow-up period may reveal different patterns of mortality risk. Future studies with extended observational periods are necessary to validate the associations observed in our cohort. Additionally, the cross-sectional design limits the ability to establish causality. The relatively small sample size may underpower comparison between the MIS and the SARC-F, limiting the generalizability of our findings. Despite adjustments for key confounders, the possibility of other unmeasured factors such as physical activity and unaccounted-for comorbidities cannot be entirely ruled out. Furthermore, the lack of objective body composition measures, due to the constraints of the COVID-19 pandemic, could lead to the overestimation of muscle abnormalities. Despite these limitations, our study underscores the critical role of the MIS in routine assessments, emphasizing its value in predicting adverse outcomes and guiding further interventions in HD patients.

## 5. Conclusions

The early recognition and timely treatment of PEW are integral components of comprehensive care for CKD patients. While the SARC-F has proven effective in assessing the functional capacity of patients with CKD, it often does not correspond to increased sarcopenia risk and clear clinical and biochemical indicators of PEW, particularly in younger, active patients with preserved functional status. For these patients, detailed tools such as the MIS are recommended to ensure the accurate identification of those at the highest risk for negative outcomes, thus enabling more effective intervention and management strategies.

## Figures and Tables

**Table 1:** Baseline patients’ characteristics.

Characteristics	All Patients (*n* = 109)
Demographic	
Gender M:F (%)	61.5:38.5%
Age (median; years)	61 (27–85)
HD vintage (median; months)	50 (6–168)
Primary kidney disease (%)	
Diabetic nephropathy	28.4%
Glomerulonephritis	27.5%
Hypertensive kidney disease	16.5%
Other	27.6%
Screening for sarcopenia and PEW	
MIS (median)	7 (5–21)
SARC-F (median)	1 (0–10)
ISRNM PEW criteria (%)	
Normal status	21.1%
Mild PEW	29.4%
Moderate PEW	23.9%
Severe PEW	5.5%
Comorbidities (%)	
Diabetes	26.9%
CVD	39%
AH	77%

**Table 2:** Comparison between non-diabetic (non-DM) and diabetic (DM) patients; *p* < 0.05 is considered statistically significant.

	Median (Interquartile Range)		R (95% CI)	*p* *
	Non-DM (*n* = 79)	DM (*n* = 30)		
Age (years)	59 (46–72)	69 (64.8–74.8)	10 (4 to 16)	0.001
HD vintage (months)	46 (24–81)	31 (14.3–47.8)	−16 (−30 to −5)	0.004
BMI (kg/m^2^)	24.26 (21.15–29.35)	28.5 (24.9–32.0)	3.26 (1.04 to 5.51)	0.006
SARC-F	0 (0–2)	3.5 (0–6)	1 (0 to 3)	0.008
MIS	7 (5–9)	9 (6–11)	2 (0 to 4)	0.01
Prealbumin (g/L)	0.31 (0.26–0.36)	0.27 (0.2–0.3)	−0.04 (−0.08 to −0.01)	0.01
Albumin (g/L)	40.4 (37.3–43)	36.5 (34.3–40.8)	−3.4 (−5.2 to −1.6)	0.001
Total protein (g/L)	67 (64–70)	64.5 (59.8–69)	−2 (−5 to 0)	0.12
Calcium (mmol/L)	2.15 (2.09–2.28)	2.1 (2–2.2)	−0.08 (−0.14 to −0.01)	0.02
Phosphorus (mmol/L)	1.8 (1.44–2.16)	1.7 (1.4–2)	−0.11 (−0.34 to 0.10)	0.26
TIBC (µmol/L)	40 (36–46)	37 (34–42)	−4 (−6 to 0)	0.02
Cholesterol (mmol/L)	3.4 (2.8–4.2)	3.5 (2.7–4.3)	0 (−0.5 to 0.4)	0.87
Triglycerides (mmol/L)	1.3 (1–2.2)	1.8 (1–2.8)	0.22 (−0.19 to 0.68)	0.29
Hemoglobin (g/L)	109 (98–116)	105 (100–114.3)	−2 (−7 to 4)	0.51
Glucose (mmol/L)	5.3 (4.7–6.6)	6.7 (5.6–9.9)	1.1 (0.5 to 2.1)	0.002
CRP (mg/L)	2.7 (1–7)	5.2 (2.7–14.2)	1.8 (0.3 to 3.6)	0.02
Potassium (mmol/L)	5.2 (4.8–5.7)	5.3 (4.8–5.9)	0.1 (−0.2 to 0.5)	0.52

* Mann–Whitney U test.

**Table 3:** Patients’ characteristics according to the SARC-F score; *p* < 0.05 is considered statistically significant.

	SARC-F (%)			*p* *
	0–3 (*n* = 80)	≥4 (*n* = 29)	All Patients (*n* = 109)	
Sex				
Male	52 (65)	15 (51.7)	67 (61.5)	0.21
Female	28 (35)	14 (48.3)	42 (38.5)	
Diabetes				
No	65 (81.3)	14 (48.3)	79 (72.5)	0.001
Yes	15 (18.8)	15 (51.7)	30 (27.5)	
MIS				
0–5	29 (36.3)	1 (3.4)	30 (27.5)	0.001
≥6	51 (63.8)	28 (96.6)	79 (72.5)	
CVD	28 (35)	14 (48)	42 (38)	0.21
AH	65 (81)	19 (65)	84 (77)	0.08
Weight reduction	16 (20)	14 (48.3)	30 (27.5)	0.003
Poor appetite	7 (8.8)	9 (31)	16 (14.7)	0.01 ^†^
ISRNM criteria				
Normal status	23 (28.8)	0	23 (21.1)	<0.001 ^†^
Mild PEW	30 (37.5)	2 (6.9)	32 (29.4)	
Moderate PEW	15 (18.8)	11 (37.9)	26 (23.9)	
Severe PEW	1 (1.3)	5 (17.2)	6 (5.5)	
* Sarcopenic obesity	11 (13.8)	11 (37.9)	22 (20.2)	
Outcome				
Negative (death)	4 (5)	3 (10.3)	7 (6.4)	0.38 ^†^
Positive	76 (95)	26 (89.7)	102 (93.6)	

* χ^2^ test; ^†^ Fisher’s exact test.

**Table 4:** A comparison between the groups divided according to the SARC-F; *p* < 0.05 is considered statistically significant.

	Median (Interquartile Range) SARC-F		R (95% CI)	*p* *
	0–3	≥4		
Age (years)	60 (47–69.75)	71 (65–79.5)	13 (7 to 19)	<0.001
HD vintage (months)	40 (21–61.5)	37 (18.5–74)	0 (−13 to 14)	>0.99
BMI (kg/m^2^)	24.2 (21.15–29.35)	27.5 (24.6–31.5)	3.18 (0.77 to 5.32)	0.01
MIS	6 (4.25–8)	10 (7–11)	3 (2 to 5)	<0.001
Prealbumin (g/L)	0.32 (0.26–0.36)	0.3 (0.2–0.3)	−0.05 (−0.09 to −0.02)	0.002
Albumin (g/L)	40.55 (37.23–43)	36.5 (34.2–40.5)	−3.3 (−5.1 to −1.5)	<0.001
Total protein (g/L)	67 (63.25–71)	65 (61.5–69)	−2 (−4 to 1)	0.18
Calcium (mmol/L)	2.16 (2.09–2.26)	2.1 (2–2.2)	−0.07 (−0.14 to −0.01)	0.03
Phosphorus (mmol/L)	1.79 (1.46–2.16)	1.6 (1.3–2.1)	−0.16 (−0.39 to 0.07)	0.17
TIBC (µmol/L)	40 (35.25–45)	39 (34–42.5)	−2 (−5 to 1)	0.27
Cholesterol (mmol/L)	3.5 (2.9–4.18)	3.4 (2.7–4.6)	−0.2 (−0.6 to 0.3)	0.37
Triglycerides (mmol/L)	1.4 (1–2.5)	1.6 (0.9–2.3)	−0.05 (−0.39 to 0.36)	0.86
Hemoglobin (g/L)	109 (101–116.8)	102 (94.5–111)	−7 (−12 to −1)	0.02
Glucose (mmol/L)	5.6 (4.8–7)	5.7 (5–7.8)	0.3 (−0.3 to 1)	0.37
CRP (mg/L)	2.8 (1–6.9)	4.6 (2.3–8.3)	1.2 (−0.1 to 2.3)	0.10
Potassium (mmol/L)	5.3 (4.9–5.9)	5 (4.7–5.7)	−0.2 (−0.6 to 0.1)	0.13

* Mann–Whitney U test.

**Table 5:** Patients’ characteristics according to the MIS; *p* < 0.05 is considered statistically significant.

	MIS (%)			*p* *
	0–5 (*n* = 47)	≥6 (*n* = 62)	All Patients (*n* = 109)	
Sex				
Male	29 (61.7)	38 (61.3)	67 (61.5)	0.97
Female	18 (38.3)	24 (38.7)	42 (38.5)	
Diabetes				
No	39 (83)	40 (64.5)	79 (72.5)	0.03
Yes	8 (17)	22 (35.5)	30 (27.5)	
SARC-F				
0–3	43 (91.5)	37 (59.7)	80 (73.4)	<0.001
≥4	4 (8.5)	25 (40.3)	29 (26.6)	
CVD	12 (25)	30 (48)	42 (38)	0.02
AH	37 (78)	47 (76)	84 (77)	0.72
Weight reduction	6 (12.8)	24 (38.7)	30 (27.5)	0.003
Poor appetite	0	16 (25.8)	16 (14.7)	<0.001
ISRNM criteria				
Normal status	22 (46.8)	1 (1.6)	23 (21.1)	<0.001 ^†^
Mild PEW	13 (27.7)	19 (30.6)	32 (29.4)	
Moderate PEW	3 (6.4)	23 (37.1)	26 (23.9)	
Severe PEW	0	6 (9.7)	6 (5.5)	
* Sarcopenic obesity	9 (19.1)	13 (21)	22 (20.2)	
Outcome				
Negative (death)	0	7 (11.3)	7 (6.4)	0.02 ^†^
Positive	47 (100)	55 (88.7)	102 (93.6)	

* χ^2^ test; ^†^ Fisher’s exact test.

**Table 6:** A comparison between the groups divided according to the MIS; *p* < 0.05 is considered statistically significant.

	Median (Interquartile Range) MIS		R (95% CI)	*p* *
	0–5	≥6		
Age (years)	57 (35–65.75)	65 (56–75)	11 (5 to 18)	0.001
HD vintage (months)	39 (20.5–57.25)	41 (20–67)	4 (−8 to 17)	0.47
BMI (kg/m^2^)	26.05 (21.22–30.57)	25.1 (22.3–29.4)	−0.98 (−3.32 to 1.57)	0.47
SARC-F	0 (0–1)	1 (0–6)	1 (0 to 2)	<0.001
Prealbumin (g/L)	0.36 (0.31–0.44)	0.3 (0.2–0.3)	−0.08 (−0.12 to −0.04)	<0.001
Albumin (g/L)	42.75 (41–44.15)	38 (35–41)	−4.7 (−6.2 to −3.2)	<0.001
Total protein (g/L)	69 (65.75–73)	65 (62–69)	−4 (−6 to −1)	0.003
Calcium (mmol/L)	2.16 (2.1–2.24)	2.1 (2–2.3)	−0.03 (−0.09 to 0.03)	0.42
Phosphorus (mmol/L)	1.88 (1.54–2.21)	1.7 (1.4–2)	−0.2 (−0.4 to 0.03)	0.08
TIBC (µmol/L)	41 (38.5–45.25)	39 (34–43)	−3 (−6 to 0)	0.02
Cholesterol (mmol/L)	3.5 (2.88–4.1)	3.4 (2.7–4.2)	−0.1 (−0.5 to 0.3)	0.74
Triglycerides (mmol/L)	1.3 (1.1–2.2)	1.6 (0.9–2.5)	−0.04 (−0.36 to 0.37)	0.86
Hemoglobin (g/L)	115 (109–119.3)	105 (97–112)	−9 (−14 to −5)	<0.001
Glucose (mmol/L)	5.3 (4.9–6.2)	5.9 (4.9–7.4)	0.4 (−0.1 to 1.1)	0.15
CRP (mg/L)	1.9 (1–6.1)	3.3 (1.6–7.3)	0.85 (0 to 2)	0.09
Potassium (mmol/L)	5.4 (4.9–6)	5.1 (4.7–5.7)	−0.3 (−0.6 to 0.1)	0.11

* Mann–Whitney U test.

**Table 7:** Predictors of negative outcome (univariate regression analysis); *p* < 0.05 is considered statistically significant.

Bivariate Analysis	ß	OR	95% CI	*p*
Gender	0.19	1.21	0.26–5.70	0.81
Age	0.04	1.04	0.98–1.11	0.19
HD vintage	−0.001	0.99	0.97–1.02	0.94
Diabetes	0.73	2.08	0.44–9.92	0.36
CVD	−0.48	0.62	0.12–3.35	0.58
AH	0.61	1.85	0.21–16.1	0.57
SARC-F	0.19	1.21	0.96–1.53	0.11
SARC-F (≥4)	0.79	2.19	0.46–10.5	0.33
BMI	−0.05	0.95	0.82–1.11	0.53
MIS	0.25	1.28	1.02–1.60	0.03
MIS (≥6)	18.9	-	-	>0.99
ISRNM criteria				
Normal status				
Mild PEW	0	1	-	>0.99
Moderate PEW	18.2	-	-	>0.99
Severe PEW	20.8	-	-	>0.99
Sarcopenic obesity	18.9	-	-	>0.99
Albumin	−0.16	0.86	0.71–1.03	0.09
Total protein	−0.05	0.95	0.86–1.06	0.36
Calcium	−2.20	0.11	0.001–24.2	0.43
Phosphorus	1.29	3.63	0.74–17.9	0.11
Cholesterol	−0.37	0.69	0.27–1.76	0.44
Triglycerides	−0.11	0.90	0.41–1.99	0.80
Hemoglobin	−0.02	0.98	0.92–1.04	0.48
Glucose	0.01	1.01	0.69–1.48	0.95
CRP	0.06	1.06	1.01–1.12	0.03
Potassium	−0.08	0.93	0.31–2.76	0.89
Weight loss	1.46	4.32	0.68–27.4	0.12
Reduced appetite	−18.4	-	-	>0.99

**Table 8:** Predicting the probability of a negative outcome (multivariate regression analysis).

	ß	OR *	95% CI	*p*
MIS	0.467	1.59	1.11–2.29	0.01
Constant	−16.64			0.03

* Adjusted for age, comorbidities (diabetes, CVD, AH), HD vintage, albumin, and CRP levels.

## Data Availability

The original contributions presented in the study are included in the article, further inquiries can be directed to the corresponding authors.
